# Rethinking the Diabetes–Cardiovascular Disease Continuum: Toward Integrated Care

**DOI:** 10.3390/jcm14186678

**Published:** 2025-09-22

**Authors:** Alfredo Caturano, Cassandra Morciano, Katarzyna Zielińska, Vincenzo Russo, Marco Alfonso Perrone, Cesare Celeste Berra, Caterina Conte

**Affiliations:** 1 Department of Human Sciences and Promotion of the Quality of Life, San Raffaele Roma University, 00166 Rome, Italy; caterina.conte@uniroma5.it; 2 Centro Malattie Endocrine e Metaboliche, Dipartimento di Scienze Mediche e Chirurgiche, Fondazione Policlinico Universitario A. Gemelli IRCCS, Università Cattolica del Sacro Cuore, 00168 Rome, Italy; morcianocassandra@gmail.com; 3 Scanmed Medical Center in Krakow, 30150 Krakow, Poland; katarzynaxzielinska@gmail.com; 4 Sbarro Institute for Cancer Research and Molecular Medicine, Center for Biotechnology, College of Science and Technology, Temple University, Philadelphia, PA 19122, USA; v.p.russo@libero.it; 5 Division of Cardiology, Department of Medical Translational Sciences, University of Campania Luigi Vanvitelli, 80138 Naples, Italy; 6 Department of Cardiology and CardioLab, University of Rome Tor Vergata, 00133 Rome, Italy; marco.perrone@uniroma2.it; 7 Department of Endocrinology and Metabolic Diseases, IRCCS MultiMedica, 20099 Milan, Italy; cesare.berra@multimedica.it

**Keywords:** cardiometabolic continuum, type 2 diabetes mellitus, cardiovascular disease, cardiovascular-kidney-metabolic syndrome, GLP-1 receptor agonists, tirzepatide, SGLT2i

## Abstract

Type 2 diabetes mellitus (T2D) and cardiovascular disease (CVD) are not merely coexisting epidemics but co-evolving manifestations of a shared cardiometabolic continuum. Despite advances in glycemic, lipid, and blood pressure control, residual cardiovascular risk remains high, underscoring the limitations of siloed approaches. In this perspective, we argue for reframing T2D and CVD as interconnected conditions driven by inflammation, adipose tissue dysfunction, and organ crosstalk. Beyond metformin, which remains foundational, several glucose-lowering drug classes are now evaluated not only for glycemic control but also for their cardiovascular and renal impact. Landmark trials and recent meta-analyses confirm that sodium-glucose co-transporter 2 inhibitors and glucagon-like peptide-1 (GLP-1) receptor agonists improve cardiorenal outcomes. More recently, tirzepatide, a dual glucose-dependent insulinotropic polypeptide (GIP)/GLP-1 receptor agonist, has shown unprecedented efficacy in weight and glucose management, with potential to further transform cardiometabolic risk reduction. Yet enthusiasm for these therapies must be tempered by heterogeneity of response, treatment costs, and inequitable access. Integrated care models, supported by multidisciplinary teams, digital health tools, and value-based reimbursement, are essential to close the gap between trial efficacy and real-world outcomes. Attention to sex, age, ethnicity, and comorbidity profiles is critical to ensure equity, as is the adaptation of strategies to low- and middle-income countries where the burden of cardiometabolic disease is rapidly rising. Ultimately, advancing cardiometabolic medicine requires not only novel therapies but also a unifying framework that integrates biology, behavior, economics, and health systems to deliver the right treatment to the right patient at the right time.

## 1. Introduction: A Converging Pathology

Cardiovascular disease (CVD) and type 2 diabetes mellitus (T2D) continue to dominate the global burden of chronic disease, each accounting for millions of deaths and extensive disability-adjusted life years [1,2]. Data from the Global Burden of Disease (GBD) 2021 show that annual CVD cases and deaths nearly doubled between 1990 and 2021, rising to 66.8 million cases and 19.4 million deaths, despite declines in age-standardized rates. In parallel, diabetes affected 529 million people globally in 2021 (age-standardized prevalence 6.1%), with T2D accounting for 96% of cases. Projections suggest that, by 2050, more than 1.3 billion people will be living with diabetes [1,2]. Their rising prevalence is not only a reflection of aging populations and sedentary lifestyles but also of increasingly complex interactions between metabolic, inflammatory, and vascular dysfunctions [3,4].

In this context, the recently defined cardiovascular–kidney–metabolic (CKM) syndrome underscores the systemic nature of these conditions, highlighting how metabolic risk factors such as obesity, dysglycemia, hypertension, and dyslipidemia interact with chronic kidney disease (CKD) and the cardiovascular system to drive multiorgan dysfunction and markedly increase the risk of adverse cardiovascular and renal outcomes [5,6]. While once studied and treated as distinct entities, the clinical and biological overlap between T2D and CVD has become undeniable. Despite remarkable advances in glycemic control, lipid-lowering strategies, and antihypertensive therapies, the dual epidemic of T2D and CVD shows no signs of retreat. Indeed, individuals with T2D remain at significantly increased risk for myocardial infarction, heart failure, stroke, and cardiovascular mortality, even when standard treatment targets are achieved [7]. This persistent risk highlights the inadequacy of siloed approaches and the need to reframe how these diseases are understood and managed.

In this perspective, we propose that T2D and CVD should not merely be considered co-occurring conditions, but rather co-evolving manifestations of a shared pathophysiological trajectory, driven by systemic inflammation, insulin resistance, adipose tissue dysfunction, and maladaptive neurohormonal responses. This shift from a dual-diagnosis model to a unified cardiometabolic paradigm has profound implications for basic science, clinical care, and public health. By exploring key mechanistic insights, therapeutic advances, and translational gaps, we aim to outline a vision for integrated, forward-thinking cardiometabolic medicine.

## 2. From Parallel Paths to a Common Road: Lessons from Trials and Models

For decades, the management of T2D focused primarily on glycemic control, under the assumption that lowering blood glucose would inevitably translate into cardiovascular benefit. This view, rooted in the legacy of trials like the UK Prospective Diabetes Study (UKPDS), emphasized microvascular protection—retinopathy, nephropathy, and neuropathy, as the primary therapeutic goals [8]. However, macrovascular complications, particularly atherosclerotic CVD and heart failure, remained stubbornly prevalent even in patients with well-controlled T2D. A series of landmark cardiovascular outcomes trials (CVOTs) over the past two decades has reshaped this paradigm. Studies such as ACCORD, ADVANCE, and VADT challenged the notion that intensive glucose lowering alone could significantly reduce major cardiovascular events [9]. In fact, evidence from ACCORD and related trials has shown that an overly intensive reduction of glycated hemoglobin (HbA1c) may be counterproductive, as it has been associated with increased risks, including hypoglycemia and even higher mortality in some subgroups [9]. This highlights the need for a balanced approach to glycemic control, emphasizing cardiovascular risk reduction rather than targeting near-normal HbA1c at all costs.

More strikingly, trials like EMPA-REG OUTCOME, LEADER, and DECLARE-TIMI 58 demonstrated that specific glucose-lowering agents, namely, sodium-glucose co-transporter 2 (SGLT2) inhibitors and glucagon-like peptide-1 receptor agonists (GLP-1 RAs), could confer cardiovascular protection independent of their glucose-lowering effects [10,11]. More recently, the DAPA-MI and EMPACT-MI trials have expanded this concept by showing that SGLT2 inhibitors may also provide significant cardioprotective effects in patients with recent myocardial infarction, even in the absence of T2D, further supporting a broader role of this drug class in cardiovascular prevention [12,13]. This marked a pivotal shift from a glucose-centric to a cardiovascular risk-centric model of T2D care. Yet, despite these advances, substantial challenges remain. Residual cardiovascular risk persists even among patients treated with the most effective agents, suggesting that our current models may not fully capture the complexity of cardiometabolic disease [14]. Fragmentation of care, where diabetologists, cardiologists, and primary care physicians operate in parallel rather than in concert, limits the integration of evidence into practice [15]. Moreover, real-world data indicate underutilization of cardioprotective therapies, especially among high-risk populations, revealing gaps between clinical trial efficacy and population-level effectiveness [16].

These limitations call for a broader, more unified framework, one that transcends traditional specialty boundaries and addresses the multifaceted drivers of cardiometabolic risk. The next evolution in care will require not only new drugs, but also new models: integrative, patient-centered, and capable of bridging the historical divide between metabolic and cardiovascular medicine. For clinicians, this paradigm shift means prioritizing therapies with proven cardiovascular benefit, screening systematically for heart failure and CKD in people living with T2D, and moving toward team-based, patient-centered care models. In practice, this requires embedding cardiometabolic risk management as a standard component of T2D care, rather than an optional add-on.

## 3. Beyond Glucose and Lipids: A Web of Shared Pathophysiology

While traditional risk factors such as hyperglycemia, dyslipidemia, obesity, and hypertension remain central in the pathogenesis of T2D and CVD, they no longer suffice to explain the magnitude and diversity of complications observed in clinical practice. Mounting evidence points to a far more intricate web of interconnected biological processes that link these conditions at multiple levels (Table 1) [17].

### 3.1. Emerging Mechanisms Beyond Traditional Risk Factors

Chronic low-grade inflammation, adipose tissue dysfunction, and vascular injury are increasingly recognized as shared drivers of both T2D and CVD [18]. Emerging factors such as inflammasome activation, gut dysbiosis, and epigenetic regulators like microRNAs illustrate the complexity of this continuum, linking metabolic dysfunction to vascular disease [19].

In the heart, processes such as fibrosis and maladaptive remodeling further emphasize that T2D and CVD are not parallel tracks but interconnected outcomes of systemic cardiometabolic stress [20,21,22]. The latest evidence also highlights the active role of epicardial adipose tissue (EAT) in this remodeling process, where the balance between white and brown-like adipocytes critically influences myocardial inflammation, oxidative stress, and coronary microvascular function [23,24]. These overlapping processes highlight the biological interconnectedness of T2D and CVD, as well as the limitations of reductionist approaches that focus on single biomarkers or isolated endpoints [25]. The clinical heterogeneity observed, from early atherothrombosis to advanced heart failure, reflects this complex and evolving pathophysiological continuum.

Acknowledging this complexity is not a call for therapeutic paralysis, but rather an invitation to refine our models. The shift toward a unified cardiometabolic framework requires a more integrative understanding of these interacting systems, one that moves beyond glucose and lipids to address the true drivers of disease progression and residual risk.

### 3.2. Organ Crosstalk and the Multisystem Basis of Cardiometabolic Disease

This network of mechanisms aligns with the concept of *organ crosstalk*, whereby metabolic, inflammatory, and neurohormonal signals are exchanged across organs to amplify cardiometabolic dysfunction [26]. For instance, the cardio–renal–metabolic axis links hyperglycemia and insulin resistance to maladaptive kidney activation of the renin–angiotensin–aldosterone system (RAAS), which in turn worsens cardiac remodeling and vascular stiffness [27]. The adipo-cardiac axis involves the secretion of pro-inflammatory adipokines and the expansion of epicardial adipose tissue that promote myocardial fibrosis and microvascular dysfunction [28]. Similarly, the hepato-cardiac axis contributes via hepatic insulin resistance, ectopic fat deposition, and altered bile acid signaling that exacerbate systemic inflammation and atherogenesis [29]. Emerging evidence also supports gut-liver and gut-cardiac pathways, with dysbiosis and microbial metabolites such as trimethylamine-N-oxide modulating endothelial and myocardial function [30]. This “organ talk” perspective helps explain why single-target approaches often fail to eliminate residual cardiovascular risk in diabetes, and why integrated strategies, simultaneously addressing multiple organ systems, are required to modify disease trajectory.

## 4. Lifestyle and Preventive Strategies

### 4.1. Physical Activity and Exercise

Physical inactivity is a well-recognized modifiable risk factor that contributes substantially to the global burden of T2D and CVD. Data from the Global Burden of Disease Study indicate that low physical activity is directly responsible for a considerable proportion of incident T2D and CVD cases [31]. Regular exercise improves insulin sensitivity, reduces visceral adiposity, lowers blood pressure, and exerts favorable effects on lipid metabolism and endothelial function [32,33]. It also modulates inflammatory pathways, thereby targeting key mechanisms common to both T2D and CVD [34]. Clinical and epidemiological studies consistently show that individuals who achieve recommended activity levels experience a lower incidence of diabetes and major cardiovascular events. Current international guidelines advocate for at least 150 min per week of moderate-intensity aerobic exercise, combined with resistance training on two or more days per week [35]. Importantly, even modest increases in physical activity among sedentary individuals confer measurable benefits, supporting the concept of a dose-response relationship [36]. Thus, exercise represents a low-cost, high-impact cornerstone of cardiometabolic prevention.

### 4.2. Nutritional Recommendations

Dietary quality exerts a profound influence on both the development and progression of T2D and CVD [37]. The Mediterranean diet, characterized by high consumption of fruits, vegetables, legumes, whole grains, nuts, olive oil, and fish, has been consistently associated with reduced incidence of diabetes and cardiovascular events [38]. Its beneficial effects are mediated by weight control, antioxidant and anti-inflammatory nutrients, improved lipid and glucose metabolism, and favorable modulation of the gut microbiota [38]. In contrast, the Western diet, rich in processed foods, red and processed meats, refined carbohydrates, saturated fats, and sugar-sweetened beverages, is strongly linked to obesity, insulin resistance, systemic inflammation, and heightened cardiovascular risk [39]. Large prospective cohorts and interventional trials, such as PREDIMED, demonstrate that adherence to a Mediterranean-like dietary pattern reduces both diabetes onset and cardiovascular morbidity [40].

### 4.3. Nutraceuticals

Alongside lifestyle and dietary strategies, several nutraceuticals have been investigated for their potential to reduce cardiometabolic risk. Plant-derived compounds such as berberine, cinnamon, banaba, seaweed polyphenols, and Gymnema sylvestre have demonstrated favorable effects on glucose metabolism by improving insulin sensitivity, enhancing GLUT4-mediated glucose uptake, and reducing hepatic gluconeogenesis. In particular, berberine has shown glucose-lowering effects comparable to metformin in some trials, with additional improvements in lipid parameters [41].

Among non-plant compounds, Coenzyme Q10 (CoQ10) has gained attention for its mitochondrial, antioxidant, and anti-inflammatory properties. Recent umbrella reviews and meta-analyses suggest that supplementation can reduce fasting plasma glucose (from −5 to −11 mg/dL) and HbA1c (from −0.1% to −1.8%), while producing modest benefits in terms of lipids, particularly triglycerides and HDL-C. These effects appear most evident in individuals with T2D or metabolic disorders, in whom CoQ10 deficiency is common. However, long-term cardiovascular outcomes remain uncertain, and variability in dosage and formulations limits firm conclusions [42]. For now, nutraceuticals should be considered adjunctive options that complement, but do not replace, established lifestyle and pharmacological therapies.

## 5. From Innovation to Integration: The Therapeutic Shift

### 5.1. Metformin: The Foundational Therapy

Metformin remains the first-line pharmacological treatment for T2D [43]. Its glucose-lowering action is primarily mediated by inhibition of hepatic gluconeogenesis and improved insulin sensitivity, with additional effects on the gut microbiota and bile acid metabolism. Clinically, metformin provides durable glycemic control, modest weight reduction, and favorable effects on lipid profiles. The UKPDS and subsequent analyses demonstrated significant reductions in cardiovascular events and all-cause mortality among patients treated with metformin, reinforcing its cardiometabolic benefits [43]. Despite being an older drug, metformin continues to serve as the cornerstone of T2D therapy.

### 5.2. GLP-1 Receptor Agonists and SGLT2 Inhibitors

The therapeutic landscape of T2D and CVD has undergone a quiet revolution. No longer confined to glycemic control alone, treatment is increasingly evaluated for its ability to modify cardiovascular and renal outcomes. At the forefront of this shift are two pharmacological classes, GLP-1 RAs and SGLT2i, which have redefined what it means to treat T2D effectively (Table 2) [44,45,46]. GLP-1 RAs, originally developed as glucose-lowering agents for their incretin effects on insulin secretion, have demonstrated consistent reductions in major adverse cardiovascular events (MACE), particularly stroke and myocardial infarction, across multiple trials [46]. A recent meta-analysis including over 85,000 participants further showed that GLP-1 RAs also reduce clinically important kidney outcomes, lowering the risk of composite kidney events by 18% and kidney failure by 16%, in addition to reducing all-cause mortality [47]. SGLT2 inhibitors, while also lowering blood glucose by promoting glucosuria independently of insulin secretion and action, have shown robust benefits in preventing heart failure hospitalization and slowing the progression of CKD, benefits that appear largely independent of their glucose-lowering action [48,49].

Recent high-quality evidence strengthens these conclusions. A living systematic review and network meta-analysis including 869 trials and 493,168 participants confirmed with moderate-to-high certainty that SGLT2 inhibitors and GLP-1 RAs reduce major cardiovascular and kidney outcomes beyond glycemic control [50]. Importantly, the analysis highlights that the absolute benefits of these therapies vary substantially depending on baseline cardiovascular and renal risk, underscoring the need for risk-stratified implementation within integrated care models. In addition, recent findings indicate that SGLT2 inhibitors may promote a favorable cardiac phenotype by reducing EAT, which contributes to lower local inflammation and improved coronary microvascular function [51]. These findings have catalyzed a rethinking of treatment goals. In today’s cardiometabolic paradigm, the question is no longer only “how low is the HbA1c?” but “what complications can we prevent?” Optimized care aims simultaneously at glycemic stability, cardiovascular protection, renal preservation, weight management, and quality of life, regardless of age [49,52]. However, real-world adoption of these paradigm-shifting therapies remains uneven.

Despite strong guideline recommendations, GLP-1 RAs and SGLT2i are underprescribed, particularly among populations most at risk: older adults, those with heart failure or CKD, and individuals from socioeconomically disadvantaged backgrounds [53,54]. Barriers include cost, therapeutic inertia, lack of familiarity among non-endocrine specialists, and fragmented care pathways that silo diabetology, cardiology, and nephrology [55,56]. Bridging the gap between clinical trial evidence and everyday practice requires more than awareness; it demands structural integration. Concrete strategies are needed to operationalize this integration. Value-based reimbursement models that reward the prevention of cardiovascular events rather than short-term metrics can align incentives across specialties [55]. The establishment of integrated cardiometabolic clinics, bringing together cardiologists, diabetologists, and nephrologists, offers a way to deliver cohesive, patient-centered care. At the population level, policies that expand access to GLP-1 RAs, SGLT2i, and emerging therapies are essential to ensure that innovation reaches those most at risk.

### 5.3. An Emerging Therapy: Tirzepatide

Among emerging therapies, tirzepatide, the first dual GIP/GLP-1 receptor agonist, represents a potential next step in cardiometabolic medicine. In clinical trials, tirzepatide has achieved unprecedented improvements in glycemic control and body weight, far exceeding most existing GLP-1 RAs [57]. Notably, its dual GIP/GLP-1 agonism may provide additional metabolic flexibility and potential anti-inflammatory benefits, beyond glucose-lowering effects. Given the central role of obesity and insulin resistance in driving both T2D and CVD, these effects may carry important cardiovascular implications [29]. This may be particularly relevant in the context of integrated cardiometabolic care, where simultaneous targeting of obesity, insulin resistance, and cardiovascular risk has emerged as a central therapeutic strategy.

Preliminary and not peer-reviewed results from the SURPASS-CVOT trial (NCT04255433, July 2025) [58] confirmed non-inferiority of tirzepatide compared with dulaglutide for MACE-3 (HR 0.92, 95.3% CI 0.83–1.01; *p* = 0.086). Tirzepatide was associated with a 16% lower risk of all-cause mortality, slower eGFR decline in people living with CKD, and greater reductions in HbA1c and body weight. Pre-specified indirect comparisons with placebo suggested substantial relative risk reductions in MACE and mortality, though these require cautious interpretation until peer-reviewed data are available. Together, these findings indicate that tirzepatide preserves the cardioprotective profile of established GLP-1 RAs while offering additional metabolic and renal advantages, positioning it as a candidate for the next generation of integrated, multi-organ therapies [58]. These findings are consistent with evidence from the most recent living systematic review and network meta-analysis, which identified tirzepatide as the most effective therapy for weight reduction among adults with type 2 diabetes (mean difference −8.63 kg versus placebo, moderate certainty), while also confirming gastrointestinal adverse events as the main safety concern [50].

### 5.4. Other Glucose-Lowering Drugs

Beyond metformin, SGLT2 inhibitors, GLP-1 receptor agonists, and emerging dual incretin therapies, other drug classes remain integral to diabetes management, though their cardiometabolic benefits are more limited. Sulfonylureas have long served as effective and inexpensive agents for lowering HbA1c, but their use is tempered by risks of hypoglycemia, weight gain, and possible adverse cardiovascular profiles with older compounds [59]. DPP-4 inhibitors offer modest glucose-lowering efficacy, are weight neutral, and generally well tolerated, yet large outcome trials have shown cardiovascular safety without additional cardioprotective benefit; furthermore, some agents (e.g., saxagliptin, alogliptin) have been linked to increased heart failure hospitalization [60]. Insulin remains the most potent therapy for lowering glucose and is indispensable in advanced T2D, catabolic presentations, and severe hyperglycemia. However, its use is complicated by hypoglycemia risk, weight gain, and the practical burden of injections and monitoring [61]. While these agents continue to play a role in individualized glycemic management, their limitations reinforce the growing preference for therapies that combine metabolic control with proven cardiovascular and renal protection (Table 3).

### 5.5. Lipid-Lowering and Cardioprotective Therapies Beyond Glucose Control

It is important to recognize that cardiometabolic care also relies on advances in lipid-lowering and cardioprotective therapies. Statins remain the cornerstone of LDL-cholesterol reduction and cardiovascular prevention, but additional agents have significantly expanded treatment options [62]. Ezetimibe provides incremental LDL-C lowering when combined with statins, while proprotein convertase subtilisin/kexin type 9 (PCSK9) inhibitors have demonstrated robust reductions in LDL-C and major adverse cardiovascular events [63,64]. More recently, inclisiran, a small interfering RNA targeting PCSK9, offers durable LDL-C lowering with biannual dosing, potentially improving adherence [65]. Bempedoic acid has also emerged as an effective option, particularly in statin-intolerant patients, with a recent meta-analysis showing significant LDL-C reduction and a decrease in major cardiovascular events [66]. Beyond LDL-C, therapies targeting hypertriglyceridemia and elevated lipoprotein(a) are in development and may further broaden cardiovascular risk management [62]. In parallel, optimization of antihypertensive strategies and RAAS blockade continues to be central [67]. Acknowledging this broader therapeutic landscape underscores that the value of integrated models lies not in privileging a single drug class, but in orchestrating multiple complementary interventions to achieve comprehensive cardiometabolic risk reduction.

## 6. Integrated Care and Inequity

Multidisciplinary care frameworks, shared decision-making tools, and equitable access programs are critical to translating therapeutic innovation into population-level benefit.

### 6.1. Examples of Integrated Models

Examples of such models already exist. Integrated cardiometabolic clinics, where cardiologists, endocrinologists, and nephrologists jointly manage patients, have demonstrated improvements in therapeutic adherence and cardiovascular outcomes. The Kaiser Permanente system in the U.S., for instance, has implemented multidisciplinary care pathways that reduced mortality for heart failure [68]. Similarly, Europe has piloted shared-care programs in which primary care physicians coordinate with specialists using digital registries and telemedicine [69]. Practical steps include embedding routine heart failure and CKD screening in diabetes visits, as performed in the SwissDiab study [70], adopting shared electronic health records across specialties, and aligning reimbursement models to reward long-term prevention rather than short-term metrics.

### 6.2. Economic Considerations

The economic dimension is equally critical: while newer therapies raise upfront costs, integrated care models demonstrate that long-term savings from avoided hospitalizations and complications can offset these expenditures. Multiple evaluations report that GLP-1 RAs and SGLT2 inhibitors are often cost-effective (or approach high value) when downstream events are considered, particularly for cardiovascular and renal outcomes. For example, cost-utility analyses project a favorable ICER (Incremental Cost-Effectiveness Ratio) for semaglutide in T2D and show that SGLT2 inhibitors deliver QALY (Quality-Adjusted Life Year) gains with event-related cost offsets; value improves further as drug prices decline [71,72,73]. In heart failure, modeling suggests dapagliflozin becomes high value below specific annual price points and could be cost-saving at lower prices, highlighting price sensitivity but meaningful offset potential from avoided admissions [74].

Looking ahead, prices are likely to moderate as exclusivity windows close and generics/biosimilars emerge in some markets: semaglutide protection dates begin to expire in 2026 in certain jurisdictions, with announcements of planned generic launches (e.g., Brazil) and payer expectations of substantial post-expiry price erosion [75,76]. U.S. and international assessments also emphasize that affordability and access can be improved via outcomes-based contracts and budget-guardrails that balance short-term budgets with long-term value, especially relevant as GLP-1 class use expands [77]. Overall, when integrated care models deploy these agents to the right patients (e.g., T2D with established CVD, HF, or CKD), the combination of event reduction and evolving pricing trends strengthens the economic case for adoption [73,74].

### 6.3. Equity Measures

Ensuring equitable implementation is equally critical. Socioeconomically disadvantaged groups, ethnic minorities, and patients in rural areas often face the greatest barriers to accessing GLP-1 RAs and SGLT2 inhibitors, despite deriving the largest potential benefit. Integrated care models can mitigate these disparities by embedding systematic screening for social determinants of health into diabetes and CVD visits, as recommended by the ADA Standards of Care 2025 [78]. The American Heart Association has also emphasized the need for implementation strategies designed through a cardiovascular health-equity lens [79]. Community health worker programs have been shown to improve engagement and glycemic outcomes in underserved populations, while language-concordant care is associated with lower cardiovascular events [80]. Moreover, telehealth platforms that provide device support and digital literacy training reduce the digital divide [81], transportation assistance lowers missed appointments for cardiometabolic follow-up [82], and food-as-medicine interventions (produce prescriptions or medically tailored meals) for patients with food insecurity [83,84]. At the policy level, equity-adjusted value-based reimbursement and reduced cost-sharing for high-value therapies have been proposed to expand uptake among high-risk populations [85]. Embedding these elements into integrated care is essential to ensure that therapeutic advances do not widen, but rather narrow, existing gaps in cardiometabolic outcomes.

While enthusiasm for integrated care is supported by observational studies and pilot programs, it is important to acknowledge that the evidence base remains heterogeneous. A 2023 meta-analysis of randomized controlled trials in sub-Saharan Africa found that integrated chronic care models produced a moderate reduction in systolic blood pressure (−4.85 mm Hg), though results were mixed for HbA_1_c, adherence, and quality of life [86]. A retrospective cohort study, from a cardiometabolic clinic in Vermont, has shown substantial improvements in prescription rates of GLP-1 RAs (63% vs. 24.6%), SGLT2 inhibitors (47.7% vs. 10.8%), HbA_1_c (−0.9% vs. −0.4%), and BMI (−2.8 kg/m^2^ vs. −0.5 kg/m^2^) over 6–18 months [87]. However, high-level RCT evidence remains limited: most insights derive from real-world implementation projects, registries, and case series [88]. While outcomes like adherence, intermediate metabolic control, and hospitalization rates appear favorable, long-term data on hard cardiovascular and renal endpoints are still lacking, underscoring the need for pragmatic trials and implementation science to strengthen the case for widespread adoption.

Another critical dimension of equity is the recognition of biological and clinical heterogeneity. Cardiometabolic outcomes and treatment responses differ significantly by sex, age, ethnicity, and comorbidity profiles [89,90]. For example, women with T2D experience disproportionately higher relative risk of cardiovascular events compared with men at diabetes diagnosis [91], while older adults and those with multimorbidity often face competing risks and greater vulnerability to polypharmacy [92]. Ethnic minority groups frequently encounter both higher baseline cardiometabolic risk and structural barriers to care, compounding disparities [93]. Integrated care models must therefore embed systematic consideration of these factors, through tailored risk assessment tools, inclusion of diverse populations in guideline development, and pragmatic trial designs that reflect real-world heterogeneity, to ensure that innovations are effective and generalizable across patient groups.

### 6.4. Global Perspective

While most published examples of integrated cardiometabolic care derive from North America and Europe, the burden of diabetes and cardiovascular disease is rising most rapidly in low- and middle-income countries (LMICs) [94]. Evidence from Asia, Latin America, and sub-Saharan Africa demonstrates both the urgency and feasibility of adapting integrated care to diverse health systems. For example, a systematic review of task-sharing interventions showed that non-physician health workers can effectively manage hypertension and diabetes in LMICs, leading to meaningful improvements in blood pressure control [95]. In India, a non-randomized controlled trial found that community health worker-led programs improved glycemic outcomes in resource-limited settings [96], while in rural Uganda, qualitative studies confirmed that task-shifting of diabetes and hypertension care to frontline workers is feasible and acceptable [97].

Integrated models combining HIV and cardiovascular risk management in sub-Saharan Africa and Asia have also demonstrated feasibility and potential scalability [98]. Region-specific reviews highlight the distinct epidemiological and health system challenges in the Asia Pacific, calling for tailored integrated approaches [99]. Collectively, these findings indicate that although implementation strategies will differ by context, the principles of multidisciplinary care, task-sharing, and equitable access are globally applicable and can guide adaptation of integrated cardiometabolic care beyond Western settings.

## 7. Future Thinking: Predictive Tools and Holistic Models

As cardiometabolic disease becomes increasingly recognized as a system-wide dysfunction rather than a set of isolated conditions, the need for predictive, personalized, and integrated care models has never been more urgent. Technological advances, especially in digital health, data analytics, and artificial intelligence, are enabling proactive care by integrating electronic health records and wearable sensor data to detect early signs of decompensation, stratify patients by near-term risk, and trigger timely interventions [100].

Artificial intelligence and machine learning algorithms have shown promise in identifying hidden patterns within clinical, imaging, and biochemical data that correlate with cardiovascular events, kidney function, or glycemic instability [101,102,103]. Similarly, wearable sensors and remote monitoring devices are enabling continuous assessment of key parameters, such as heart rate variability, glucose trends, and physical activity, offering the potential for early alerts, which might also be useful in case more aggressive therapies are needed, and real-time behavioral feedback [104,105,106,107].

Telemedicine platforms can integrate these technologies into routine care by enabling continuous patient-clinician communication, timely therapy adjustments, and multidisciplinary coordination. During the COVID-19 pandemic, telemedicine proved effective in maintaining glycemic control and cardiovascular follow-up, and its ongoing use could enhance access, particularly for patients in underserved areas [108]. When combined with personalized cardiometabolic profiling (e.g., genomics, metabolomics, microbiome analysis), these tools may allow for a shift from population-based guidelines to individualized prevention strategies [109,110]. However, precision medicine cannot succeed in a fragmented system. The future of cardiometabolic care must also be system-level, anchored in prevention, lifestyle modification, and equitable access to innovation [111,112]. This includes addressing structural barriers such as food insecurity, sedentary environments, and unequal access to healthcare resources [113]. It also means recalibrating payment models to reward long-term outcomes rather than short-term metrics and embedding lifestyle and behavioral counseling into routine care, not as an adjunct, but as a clinical cornerstone [114,115].

Above all, we need new care frameworks that reflect the biological reality: that T2D, CVD, kidney disease, and obesity are not distinct entities but interwoven manifestations of the same metabolic continuum. If we are to change the trajectory of the T2D-CVD epidemic, we must look beyond glucose and plaques, beyond the clinic and the prescription pad. The next frontier lies in combining biological insights, advanced digital tools, and therapeutic innovation into a unified, holistic model of cardiometabolic health.

## 8. Conclusions: Breaking the Silos

The long-standing separation of T2D and CVD in clinical care, research, and policy no longer reflects the biological truth. These are not merely comorbidities that happen to co-exist; they are shared expressions of a deeper, systemic dysfunction involving metabolism, inflammation, vascular integrity, and organ crosstalk. Treating them in isolation has yielded incremental gains at best, and persistent residual risk at worst.

A new paradigm is needed, one that views cardiometabolic disease as a unified continuum. This demands a transdisciplinary approach: integrating molecular insights with clinical acumen, leveraging digital tools alongside behavioral strategies, and translating scientific advances into policies that prioritize prevention, equity, and long-term well-being. From bench to bedside to community, the call for integration is no longer optional; it is a scientific necessity, a clinical responsibility, and an ethical imperative. As we stand at the intersection of biological complexity and therapeutic possibility, the most impactful innovations may not be in molecules or machines, but in the frameworks we build to connect them, for the whole patient, not just their parts.

GLP-1 RAs, SGLT2 inhibitors, and tirzepatide have emerged as cardiometabolic therapies that extend benefits beyond glucose lowering to cardiovascular and renal protection. It is important to acknowledge that enthusiasm for these agents must be tempered by unresolved questions. These include heterogeneity of response, high cost, inequitable access, and the absence of definitive cardiovascular outcomes data for newer agents. Furthermore, integrated care must explicitly account for heterogeneity across sex, age, ethnicity, and comorbidities, recognizing that “one-size-fits-all” approaches risk perpetuating gaps in outcomes. Personalized strategies and equitable implementation are essential to ensure that therapeutic advances translate into benefits for all subgroups.

Recognizing these limitations is critical to maintaining scientific rigor while advancing integrated care models. Ultimately, the transformative potential of the novel cardiometabolic therapies will only be realized if health systems evolve to deliver them equitably and in an integrated manner. In this context, predictive tools and molecular profiling will be key to enabling truly personalized therapies, ensuring the right treatment for the right patient at the right time. Although many illustrative programs come from North America and Europe, the principles of integrated and equitable cardiometabolic care are globally relevant, and their successful implementation will require adaptation to diverse health system contexts. Looking ahead, the growing recognition of the cardiometabolic continuum may justify dedicated thematic initiatives and collaborative research agendas, fostering a deeper understanding of shared biological mechanisms and accelerating preventive strategies across populations.

## Figures and Tables

**Table 1:** Traditional and emerging shared risk factors between cardiovascular disease and type 2 diabetes.

Category	Traditional (Established) Risk Factors	Emerging (Novel) Risk Factors
Metabolic	Hyperglycemia, Dyslipidemia (↑ LDL-C, ↓ HDL-C, ↑ TG, ↑ TC), Obesity	Insulin resistance, Adipose tissue dysfunction (visceral ectopic fat, EAT), LDL-ox, adipokines
Vascular	Hypertension, Endothelial dysfunction	Arterial stiffness, Microvascular rarefaction
Inflammatory	CRP elevation (as a marker), Chronic low-grade inflammation	NLRP3 inflammasome activation, IL-6, TNF-α (as markers)
Thrombotic	Platelet activity	Coagulation cascade imbalance, Endothelial-to-mesenchymal transition
Genetic/Epigenetic	Family history	miRNAs, SNPs affecting metabolic and vascular genes
Organ Crosstalk	—	Adipo-cardiac axis, Hepato-metabolic signaling
Microbiota-Derived	—	Gut microbiota dysbiosis, TMAO, SCFA imbalance, endotoxemia
Fibrotic/Structural	—	Myocardial fibrosis, Epicardial fat expansion
Residual Risk Factors	Post-treatment LDL, HbA1c control, Microalbuminuria	Elevated Lp(a), Inflammation despite target achievement, High-sensitivity cardiac troponin, Natriuretic peptides (BNP/NT-proBNP)

LDL low-density lipoprotein, HDL high-density lipoprotein, TG triglycerides, TC total cholesterol, OX oxidized, EAT epicardial adipose tissue, CRP C-reactive protein, Lp(a) lipoprotein(a), SNPs single nucleotide polymorphisms, miRNAs microRNAs, TMAO trimethylamine-N-oxide, SCFA short-chain fatty acids, BNP brain natriuretic peptide, NT-proBNP N-terminal pro-B-type natriuretic peptide.

**Table 2:** Translational gaps between clinical evidence and real-world and potential benchmarks.

Category	Evidence from Trials	Real-World Barriers	Potential Benchmarks
Therapeutics	SGLT2i, GLP-1 RA, tirzepatide reduce MACE, HF, CKD	Low adoption, cost/access	≥70% of eligible high-risk patients prescribed guideline-recommended agents within 12 months
Risk Stratification	CV risk calculators, renal markers	Underused, lack of personalization	Routine HF and CKD screening in ≥80% of T2D visits
Preventive Frameworks	Lifestyle + pharmacotherapy reduce progression	Limited access to counseling, socioeconomic gap	Documentation of nutrition/exercise counseling in ≥75% of clinic visits
Interdisciplinary Care	Multispecialty teams improve outcomes	Siloed care, fragmented reimbursement	Implementation of structured cardiometabolic clinics or pathways across ≥50% of health systems

MACE major adverse cardiovascular events, HF heart failure, CKD chronic kidney disease, CV cardiovascular, SGLT2i sodium-glucose co-transporter 2 inhibitors, GLP-1 RA glucagon-like peptide-1 receptor agonists.

**Table 3:** Summary of key benefits, risks, and contraindications of major glucose-lowering drug classes relevant to cardiometabolic care.

Drug Class	Key Benefits	Main Risks/Adverse Events	Contraindications
Metformin	↓ HbA1c (1–1.5%), modest weight loss, durable glycemic effect, ↓ CVD events (UKPDS), low cost	GI intolerance, vitamin B12 deficiency (long-term), rare lactic acidosis	eGFR < 30 mL/min/1.73 m^2^, advanced liver disease, severe hypoxia, alcohol abuse
Sulfonylureas	↓ HbA1c (1–1.5%), inexpensive, widely available	Hypoglycemia, weight gain, possible ↑ CV risk (older agents)	History of severe hypoglycemia, caution in elderly/frail
DPP-4 inhibitors	Modest ↓ HbA1c (0.5–0.8%), weight neutral, well tolerated	Rare pancreatitis, joint pain, possible ↑ HF hospitalization (saxagliptin, alogliptin)	History of pancreatitis, avoid saxagliptin/alogliptin in HF
Insulin	Most potent glucose-lowering (no dose ceiling), improves catabolic symptoms	Hypoglycemia, weight gain, injection burden	Hypoglycemia unawareness (relative), caution in frail elderly
SGLT2 inhibitors	↓ HbA1c (0.5–1%), weight loss, ↓ HF hospitalization, ↓ CKD progression, ↓ CV mortality	Genital infections, volume depletion, euglycemic DKA, rare amputations/fractures (mainly reported with canagliflozin in high-risk patients; controversies persist)	Type 1 diabetes, recurrent DKA, severe dehydration
GLP-1 receptor agonists	↓ HbA1c (1–1.5%), significant weight loss, ↓ MACE, ↓ kidney outcomes	GI intolerance, gallbladder disease, rare pancreatitis, thyroid C-cell tumors in rodents	Personal/family history of MTC or MEN2, severe gastroparesis
Tirzepatide (GIP/GLP-1 RA)	Greater ↓ HbA1c and weight than GLP-1 alone, possible renal/CV benefit (emerging)	GI intolerance (higher than GLP-1 RA), potential hypoglycemia with insulin/SU	Same as GLP-1 RA (MTC, MEN2), caution in severe GI disease

HbA1c glycated hemoglobin, CVD cardiovascular disease, eGFR estimated glomerular filtration rate, HF heart failure, CKD chronic kidney disease, DKA diabetic ketoacidosis, MACE major adverse cardiovascular events, MTC medullary thyroid carcinoma, MEN2 multiple endocrine neoplasia type 2, SU sulfonylurea.

## Data Availability

No dataset was generated for the publication of this article.
